# Death cafés as a strategy to foster compassionate communities: Contributions for death and grief literacy

**DOI:** 10.3389/fpsyg.2022.986031

**Published:** 2022-08-02

**Authors:** Carlos Laranjeira, Maria Anjos Dixe, Ana Querido, Jennifer Moran Stritch

**Affiliations:** ^1^School of Health Sciences of Polytechnic of Leiria, Leiria, Portugal; ^2^Centre for Innovative Care and Health Technology (ciTechCare), Leiria, Portugal; ^3^Research in Education and Community Intervention (RECI), Piaget Institute, Viseu, Portugal; ^4^Center for Health Technology and Services Research (CINTESIS), NursID, University of Porto, Porto, Portugal; ^5^Department of Applied Social Sciences, Social Sciences ConneXions Research Institute, Technological University of the Shannon, Limerick, Ireland

**Keywords:** death literacy, COVID-19 pandemic, compassionate communities, death cafés, bereavement, grief literacy

## Introduction

The death-positive movement, the most recent manifestation of the death awareness movement, contends that modern society is suffering from a “death taboo” and that people should talk more openly about death (Koksvik and Richards, 2021). This movement is striving to shift the dialogue about (and place of) death and dying into community spaces (Breen, 2020).

People are dying at older ages and over longer periods of time, as a result of chronic disease trajectories and advances in medical interventions, generating new demographic, and epidemiological trends. In many circumstances, death and dying processes are over-medicalized due to aggressive treatments and practices in hospitals and residential eldercare facilities (Becker et al., 2014). Most deaths happen within such institutions, leaving communities frequently “in the dark” regarding processes of care and illness at the end of life (Breen, 2020). There is a widespread belief that community-based solutions in palliative care and support for the bereaved are needed (Richards et al., 2020). However, as argued by Park et al. (2022), scant attention has been given to community-level interventions for death, dying and grief, or to the public's readiness to fully participate in these interventions.

The COVID-19 pandemic highlighted the global need for communities to be prepared for illness, death and grieving. As a result, the general population and health and social care professionals became keenly aware of a variety of issues connected to mortality and the end-of-life, challenging tendencies to avoid discussions about death and dying. Two related concepts attempt to counteract this reluctance to consider or discuss death: death literacy and grief literacy.

Death literacy is defined as a set of skills and knowledge enabling people to learn about, understand, and act on end-of-life and death-care options (Noonan et al., 2016). People and groups with a high level of death literacy have a context-specific comprehension of the death system and can more easily adapt to it, becoming better equipped to provide care for others or to gain access to critical services necessary for high-quality end-of-life support (Noonan et al., 2016).

Grieving often accompanies loss, which is typically, but not always, tied to death. Grief literacy has been defined as knowledge, skills, and values that promote compassion for self and others in the face of loss (Breen et al., 2022). The aims of the grief literacy movement are to understand and normalize grief, improving everyone's ability to recognize and effectively respond to loss. Increased public awareness of loss will organically encourage greater emotional and practical support around grief, with clear benefits for the bereaved as well as frontline health and social care professionals, who often have limited time and resources to sufficiently attend to these issues. Grief literacy can be viewed as a natural extension of death literacy, and increasing individual capacities in both areas may help reduce the effects of death avoidance and death anxiety (McClatchey and King, 2015).

One approach that may enhance both death and grief literacies is the Death Café. Inspired by the work of Crettaz (2010), Death Cafes are a global social franchise, with locally-organized public events that encourage discussion of all aspects of death, dying and mortality. These discussion events, often taking place in cafes, restaurants, libraries or other public spaces and facilitated by volunteer hosts, adhere to one vital rule: cake or other culturally-appropriate celebratory foods should be enjoyed by participants to honor our precious “finite lives” (Death Café, 2022). The Death Café approach, currently used in more than 81 countries worldwide, allows individuals to discuss difficult topics, increasing their self-awareness, potentially reducing death anxiety, and augmenting compassionate connections through conversation and personal sharing (Miles and Corr, 2015; Fong, 2017; Chang, 2021).

We argue that the Death Café approach can be a useful strategy to improve both death and grief literacy levels and may help promote the burgeoning concept of compassionate communities as part of palliative care (Graham-Wisener et al., 2022).

## Death cafés and compassionate communities

The Death Café movement, through its promotion of open discussion about the human realities of death and grief, has strong links to the compassionate community model. Public Health Palliative Care International (Compassionate Cities, 2022) defines a compassionate community as a community development initiative associated with global palliative care. A compassionate community consists of groups of neighbors/members of a community who come together to help people in their network living with a terminal illness, along with caregivers and the bereaved. Compassionate communities attempt to follow the 95% rule, wherein the dying person spends only 5% of end-of-life with formal medical services and the remaining time with their caregivers and connections in the community (Kellehear, 2013). Based on the premise that death and dying are shared communal concerns, care networks are created from within the existing community to support its members. Compassionate communities are framed as a public health response to death and bereavement, raising public awareness, and preparing people for death by discussing their wishes in advance and fostering compassionate behaviors toward those in the community who are nearing death or have recently experienced bereavement (Koksvik and Richards, 2021). Innovative community-based strategies, such as Death Cafes, may appeal to a wide variety of groups within the population, and can be adjusted to fit local culture and practices. The Death Café model allows for informal sharing of intimate stories and experiences within a social and convivial atmosphere (Leland, 2018). These kinds of activities may help community members better engage with the Compassionate Communities ethos (Liu et al., 2022).

Kellehear (2020) developed a model of person-centered care and the concept of Compassionate Cities for end-of-life patients. The person is surrounded by circles of care, namely (outward from the center): a closer internal network; supported by a wider external network; then the community, social and health services; and, lastly, the local and national authorities. When the Compassionate Community model is followed, family members and caregivers are more resilient and less exhausted during an individual's final days of life, and the dying person can have a better quality of life at home (Librada-Flores et al., 2020). Community support for patients and their caregivers is performed through tasks, such as spending time with the patient (e.g., reading a book, talking) or providing direct care, thus allowing the informal caregiver to rest or perform tasks outside their caregiver role. The care network may also assist by completing household chores, such as cooking, cleaning the house, pet care, shopping, etc. The idea is to create an intentional, but natural, external support network that enhances the direct care system for the individual (Librada-Flores et al., 2020). This intentional network, forming the circles of care around the dying and the bereaved, requires a high degree of comfort with intimacy and trust in the ability of community members to both offer and accept care from non-professionals during a vulnerable time in life. We believe that the personal sharing and interaction that takes place at Death Café events can help build the foundations of compassion and comfort that, over time, will allow this exchange of care to occur more naturally in groups and communities.

More compassionate connections within a community, augmented by initiatives like Death Cafes and Compassionate Communities strategies, should result in a better quality of life and death for all (Richards et al., 2020). Darwin suggested morality as the defining feature in the human-animal divide, and it can be argued that compassion within a human community is a powerful variable in survival (Wilson, 2019). Hypothetically, a person integrated into a more compassionate and generous community may survive longer and thrive within a high-quality social system. Opportunities to build compassion, and understanding and openness about death and grief within heterogeneous and constantly-evolving community structures are crucial to improving end-of-life and bereavement care for all (Abel, 2018).

### Death cafes: Sharing stories and conversations about loss, death, and grieving

The Death Café model encourages the relaxed mutual sharing of personal memories, thoughts, beliefs, and feelings about mortality and grief (Chang, 2021). Participants tell stories about their experiences of losing loved ones and funerals they have attended, anticipate their own deaths, discuss preferences for their own death, beliefs in the afterlife and many other aspects of death, dying, and bereavement. Recalling these experiences, articulating a narrative from them, and listening to the narratives of others is a unique compassion-building benefit of Death Cafes (Mitchell et al., 2021).

According to Mroz et al. (2020), narrating adverse life situations within a supportive community is associated with increased subjective well-being and resilience. Following a loss, facing the experience may imply both positive and negative reframing, and the ability to integrate these experiences into one's life story will depend on how they are remembered (Neimeyer, 2001; Mroz et al., 2020). Individuals often retrospectively reconcile the stress of loss by retelling events that emphasize personal progress (Mroz and Bluck, 2018) and communion with others. We argue that participating in Death Cafes allows the sharing of life experiences connected with dying and grief, that can contribute to this increased sense of well-being. Narratives about loss tend to include more references to personal connections than those about other life issues, particularly fond memories and recollections of intimacy with a dying or deceased loved one (Bluck et al., 2008). Furthermore, recalling personal interactions with those involved throughout the process of loss can alleviate feelings of isolation (Mroz and Bluck, 2018, 2019).

The abrupt and unexpected death of a loved one can be extremely difficult for the bereaved, and this context of death is relevant given the present pandemic scenario (Gesi et al., 2020; Morris et al., 2020). Previous research has indicated that dealing with fatalities that are discordant with the natural life cycle, such as those caused by an accident or a sudden illness, is severely challenging (Shear, 2012; Keyes et al., 2014). The unexpected death of a loved one may worsen the sense of meaninglessness that bereavement can bring and heighten existential anxiety (Tang and Xiang, 2021). Due to isolation and social distancing during the pandemic, patients dying from COVID-19 had limited physical contact at their bedside and restricted emotional comfort and consolation at end-of-life, while the bereaved endured a lack of access to, or absence of, conventional culturally-acceptable rites and social or community resources (Cardoso et al., 2020; Laranjeira and Querido, 2021; Petry et al., 2021). These restrictions affected the dying person's care options, and fostered feelings of regret among family members, who missed the opportunity to “be there” in those final moments (Breen, 2020; Wallace et al., 2020; Laranjeira et al., 2022). By emphasizing personal sharing in an informal social environment, Death Cafes offer a space for people to talk about death and mortality and process the unprecedented challenges encountered during COVID-19. Recently, there has been a surge of interest in narrative strategies for dealing with loss (Rolbiecki et al., 2021). Self-narrative framing around loss connects significant events and data into a series of components that span time and create a personal story (Ratcliffe and Byrne, 2022). Storytelling is what makes these events and facts intelligible; it gives them a function, place, and meaning, establishing the order of events in the past, present, and anticipated future. Death Cafes can offer this meaning-building activity of mutual storytelling and sharing at the community level.

We argue that death and grief literacies can better emerge within a population that engages with the Compassionate Communities concept and with social experiences such as Death Cafes. In the Death Café model, a person can remember and maintain a connection with a deceased loved one and share stories with other participants that can instill a positive reflection upon their own life, i.e., gaining a sense of personal growth, avoiding rumination, and focusing on positive social connection (Mroz et al., 2020). Therefore, Death Cafes are arguably aligned with both death and grief literacy and Compassionate Community efforts.

The pandemic has drawn attention to the need to develop grief literate societies and compassionate communities (Table 1). Although there is no single solution for assisting someone who is grieving, we provided some tips that may allow for mutual understandings and interdependent support both in the bereaved's day-to-day settings, as well as in broader society (Fang and Comery, 2021; Breen et al., 2022).

**Table 1:** Strategies to promote grief literate societies and compassionate communities (Bartone et al., 2019; Breen et al., 2022; Hasson et al., 2022).

• Solidarity actions, such as illuminating landmarks during significant cultural or religious occasions.
• Dedicated support groups for grieving individuals and families by faith communities and social groups.
• Develop awareness on the nuances of grief among health and social professionals, so they can provide appropriate health education and, if necessary, refer patients to psychosocial care.
• Incorporate psychosocial education about loss, grief, and bereavement into employee wellness programs, providing additional opportunities to recognize and talk about loss.
• Broadcast stories of loss, anguish, and grief in the media, as well as stories of hope, healing, and recovery.
• Increase opportunities for creative exploration of grief, emphasizing an art and health approach to death, dying, bereavement and grief.
• Establish universal teaching and learning about death and grief at all educational levels, from primary school to university and adult education (lifelong learning).
• Consider the creation and implementation of a national, European or International Day of Grieving and Commemoration to acknowledge grief and raise global public awareness of death and grief literacy.
• Give special emphasis to unique contexts of death and grieving, including suicide, overdose, homicide, neonatal loss, miscarriage, stillbirth, etc.
• Create more opportunities for individuals to identify what kinds of support they would want at the end-of-life and during periods of grief. For example, advance healthcare directives can assist in promoting discussions around end-of-life care and bereavement.

## Final remarks

Community-level interventions are a crucial component of a multifaceted public health strategy regarding end-of-life, particularly given the increasing challenges imposed by the changing demography of death and COVID-19. Promoting death and grief literacy through education, health promotion, and community development strategies is essential to attain the skills and culturally appropriate values for a compassionate community. Achieving improved levels of these literacies for both the general public and health and social care professionals is a process that should be prioritized.

Researchers, health professionals, and social educators must collaborate with communities in the design of death and grief literacy projects. Rather than focusing on individual-level acute grieving, initiatives like Death Cafés can promote community-wide literacy around all facets of death and loss. We suggest a dual approach: developing specialist resources while also investing in community capacity to understand grief, give empathetic care and reduce the stigma of death and bereavement.

Finally, there can be unforeseen consequences of making bereavement care a societal responsibility. Presently existing services in government, religious, and charitable sectors may decide to dispense with grief care, if they view the community as the sole source of such assistance. Ultimately, effective joint procedures and true community partnerships among professionals and the public are vital to developing death and grief literacy for all.

## Author contributions

All authors listed have made a substantial, direct, and intellectual contribution to the work and approved it for publication.

## Funding

This work was funded by national funds through FCT – Fundação para a Ciência e a Tecnologia, I.P (UIDB/05704/2020 and UIDP/05704/2020) and under the Scientific Employment Stimulus—Institutional Call—[CEECINST/00051/2018].

## Conflict of interest

The authors declare that the research was conducted in the absence of any commercial or financial relationships that could be construed as a potential conflict of interest.

## Publisher's note

All claims expressed in this article are solely those of the authors and do not necessarily represent those of their affiliated organizations, or those of the publisher, the editors and the reviewers. Any product that may be evaluated in this article, or claim that may be made by its manufacturer, is not guaranteed or endorsed by the publisher.
